# Factors Associated With Mortality in Leukemia and Lymphoma With COVID-19: A National Inpatient Sample Analysis (2020–2021)

**DOI:** 10.7759/cureus.86534

**Published:** 2025-06-22

**Authors:** Saketh Palasamudram Shekar, Barath Prashanth Sivasubramanian, Diviya Bharathi Ravikumar, Husna Qadeer, Ibthisam Ismail Sharieff, Rhea Prasad, Sindhu Chandra Pokhriyal, Amulya Bellamkonda, Mamtha Balla, Rutul Dalal

**Affiliations:** 1 Interventional Pulmonology, Pulmonary and Sleep Associates of Huntsville, Huntsville, USA; 2 Internal Medicine, Northeast Georgia Medical Center, Gainesville, USA; 3 Internal Medicine, Employees' State Insurance Corporation (ESIC) Medical College and Postgraduate Institute of Medical Science and Research, Chennai, IND; 4 Internal Medicine, One Brooklyn Health, Interfaith Medical Center, Brooklyn, USA; 5 Hematology and Oncology, Brookdale University Hospital Medical Center, Brooklyn, USA; 6 Internal Medicine, The University of Toledo, Toledo, USA; 7 Infectious Diseases, Penn State Health Eastern Region, Penn State Health St. Joseph Medical Center, Reading, USA

**Keywords:** covid-19, hematological malignancy, hsct, immunocompromised, leukemia, lymphoma, mortality, trends

## Abstract

Background

Patients with hematological malignancies face a substantially increased mortality from COVID-19. Although the peak of the COVID-19 pandemic has passed, the virus remains common, and understanding its impact on vulnerable groups such as those with hematologic malignancies remains crucial. Limited research exists on mortality patterns in leukemia and lymphoma patients during the pandemic. Studying these outcomes provides important insights into how different waves of COVID-19 affected immunocompromised individuals and supports the development of strategies for prevention, patient care, and risk reduction, which are essential both for managing emerging variants and preventing future pandemics. We aimed to identify the mortality risk of COVID-19 in leukemia (LekCov-19) and lymphoma (LymCov-19) in the United States and the mortality trends between each quartile from 2020 to 2021.

Methods

We analyzed the National Inpatient Sample database (2020-2021) to include adults (≥18 years) having leukemia and lymphoma admitted for COVID-19. Descriptive analysis, propensity matching, and multivariate regression were performed, with the p-value at ≤0.05 considered statistically significant. The risk of mortality was compared between each outcome.

Results

Among 8,191 LekCov-19 patients, 1,200 (14.7%) experienced mortality, whereas 507 out of 2,578 LymCov-19 patients (19.7%) experienced mortality. Multivariate regression showed a two-fold increase in mortality among LekCOV-19 and LymCOV-19 (p < 0.001). In both cohorts, several organ dysfunctions, including pulmonary, renal, and cardiac dysfunctions, were associated with increased mortality (p ≤ 0.0001). Similarly, in both cohorts, signs of bone marrow dysfunction, such as pancytopenia and thrombocytopenia, showed increased odds of mortality (p < 0.01). Both cohorts exhibited varying mortality trends, peaking during October-December 2020, January-March, July-September, and October-December 2021 (p ≤ 0.01). Hematopoietic stem cell transplantation recipients had lower odds of mortality in both cohorts, but did not attain statistical significance (p > 0.05).

Conclusion

COVID-19 was associated with increased mortality in leukemia and lymphoma patients. Surges in COVID-19-related mortality were identified from October 2020 to March 2021, and these trends could be pivotal in forecasting future mortality risks in cancer patients. Clinicians should refine treatment regimens and prioritize advancing clinical trials to address the effects of COVID-19 and the multiple comorbidities associated with hematological malignancies.

## Introduction

Cancer patients are more vulnerable to acquiring SARS-CoV-2 infection compared to the general population (p = 0.01) [1]. They also face a higher risk of developing severe illness due to a profoundly compromised immune system induced by both chemotherapy and the malignancy itself [1]. Notably, in a study by Lunski et al., patients with active cancer, when infected with COVID-19, had a higher mortality rate (21.2%) when compared with non-cancer patients (8.7%) (p < 0.001) [2]. It is essential to recognize that the immune status of patients can be a crucial factor contributing to the severity of complications and subsequent mortality [3]. COVID-19 infection triggers the release of excess cytokines or proinflammatory proteins (IL-1, IL-6, tumor necrosis factor-alpha (TNF-ɑ), interferon (IFN)-gamma, etc.) by the immune system. This cytokine storm and inflammation are responsible for severe consequences in multiple organs, leading to multi-organ dysfunction [4,5].

The overall mortality associated with COVID-19 was notably high in patients with hematological malignancies, ranging between 34% and 36.5%, which is significantly higher than the 23.8% mortality rate observed in patients with solid tumors [6]. Of note, a recent study in Europe observed the highest COVID-19-related mortality with myelodysplastic syndrome (42.3%), acute myeloid leukemia (40%), and hairy cell leukemia (34.8%) [7]. Factors contributing to this increased mortality risk include significant humoral and cellular immunosuppression [8], aggravated by chemotherapy [9]. Disease-related factors such as prolonged shedding and persistent immune dysregulation can lead to the exhaustion of T cells and decreased viral clearance, which play a significant role in disease progression [10]. In a recent study of patients with acute myeloid leukemia (AML), it was found that older age (p = 0.012), active disease (p < 0.001), and treatment discontinuation (p < 0.001) were associated with increased mortality [11]. Similarly, factors associated with higher mortality risk in lymphoma were age over 70 years, heart disease, and chronic kidney disease (p < 0.05) [12].

There has been a limited number of case series documenting the COVID-19 infection in individuals with hematologic malignancies, and these reports only offer limited information concerning the disease status or histological classification [13]. Most studies are limited by their narrow inclusion criteria, small sample sizes, and case duplications [14]. Many cohort studies during the early stages of the pandemic overestimated the risk of COVID-19 by including asymptomatic patients and those with a secondary diagnosis of COVID-19 [15]. Data on COVID-19’s impact on leukemia patients remain limited [16], with lymphoma patients even more underrepresented [17].

This retrospective cross-sectional study aims to assess risk factors for in-hospital mortality in leukemia and lymphoma patients primarily admitted for COVID-19 in the United States. We also aimed to examine trends in mortality from COVID-19 in leukemia and lymphoma patients from April 2020 to December 2021.

## Materials and methods

Design and data source

We performed a retrospective observational study using the 2020-2021 National Inpatient Sample (NIS) database. The NIS database is developed by the Healthcare Cost and Utilization Project (HCUP) and sponsored by the Agency for Healthcare Research and Quality (AHRQ). The NIS generates regional and national estimates of inpatient admissions and records of discharge outcomes [18]. The document encompasses various components like patient demographics (such as age, sex, race, and median income based on location), diagnosis, and procedure codes derived from the International Classification of Diseases, Tenth Revision, Clinical Modification/Procedure Coding System (ICD-10-CM/PCS). The ICD-10-CM/PCS measures severity and comorbidity, hospital characteristics, discharge status, and length of stay (LOS).

Ethical consideration, sample size, and study population

The NIS is a de-identified, publicly accessible database, and this investigation did not require institutional review board approval. This study did not involve the estimation of a predetermined sample size. The research did not employ any sampling methodology. Individuals aged 18 years or above admitted with an ICD-10-CM code of leukemia and lymphoma were included. We used a previously established method to identify patients admitted for COVID-19 infection with a history of hematological malignancies (leukemia and lymphoma) [19-22]. We compared the COVID-19 cohort with all other admissions. ICD-10-CM/PCS codes were used to identify patients and their comorbidities (acute respiratory failure, sepsis, acute kidney injury, myocardial infarction) are provided in Supplementary Table A1. Before accessing the NIS database, we ensured compliance with the data user agreement outlined by the AHRQ. Furthermore, the databases utilized comply with the Health Insurance Portability and Accountability Act (HIPAA) Privacy Rule’s definition of limited datasets and exclude explicit patient identifiers.

Outcomes

Our primary objective was to assess mortality risk in adults with leukemia or lymphoma admitted for COVID-19. The secondary objective was to evaluate the risk of mortality in the subset of this population treated with hematopoietic stem cell transplantation (HSCT).

Statistical methods

We conducted our analysis by examining continuous variables through means and t-tests, and qualitative variables were assessed using the chi-square test. We set a significance level of p ≤ 0.05. Stata v18 (StataCorp LLC, College Station, TX) was used to conduct the analysis. We used two methods to adjust for confounders: propensity matching and multivariate regression analysis. A non-parsimonious multivariate logistic regression model was developed to estimate the propensity score for COVID-19 and mortality using acute respiratory failure and invasive ventilation. The double robust method was then used to generate treatment weights, and the inverse probability of treatment weighting was used to match cases with controls using generalized linear models [23]. Multivariable regression models were built by including all the confounders that were significantly associated with the outcome on univariable analysis with a cutoff p = 0.3. Variables found to be important based on the literature were also forced into the models. Mortality trends in the patients were analyzed using univariate analysis and categorized into quartiles.

## Results

Leukemia with COVID-19

Of 8,191 Leukemia patients with COVID-19, 1,200 (14.7%) had mortality. Mortality was common in patients over 65 years of age compared to survivors (p < 0.0001). There was no difference in mortality according to gender (p > 0.05). Race was significantly associated with mortality (p < 0.01). Table 1 presents the sociodemographic characteristics of leukemia patients with COVID-19.

**Table 1:** Sociodemographic characteristics of leukemia and lymphoma patients with COVID-19. HMO: Health Maintenance Organization.

Leukemia patients with COVID-19				Lymphoma patients with COVID-19		
	Survival percentage (%) (n = 6991)	Mortality percentage (%) (n = 1200)	p-value	Survival percentage (%) (n = 2071)	Mortality percentage (%) (n = 507)	p-value
Age over 65 years	55.9	78.0	<0.0001	62.5	74.6	<0.0001
Gender			0.0741			0.3307
Male	56.7	59.5		58.4	60.8	
Female	43.3	40.5		41.6	39.3	
Race			0.0021			0.654
White	60.7	66.9		76.1	78.8	
Black	16.2	14.8		8.0	7.1	
Hispanic	16.7	13.1		11.1	8.9	
Asian or Pacific Islander	2.4	1.6		2.1	2.0	
Native American	<1	<1		<1	<1	
Other	3.4	3.1		2.5	2.8	
Median household			0.3384			0.5524
$1 - $49,999	29.0	31.0		24.4	25.5	
$50,000 - $64,999	27.5	25.4		28.3	29.1	
$65,000 - $85,999	24.5	23.8		26.0	27.1	
$86,000 or more	19.0	19.8		21.3	18.4	
Primary expected payer			<0.0001			0.0258
Medicare	54.0	71.4		60.8	67.3	
Medicaid	11.7	6.5		7.6	4.8	
Private, including HMO	26.9	17.5		27.1	22.6	
Self-pay	3.1	1.3		1.4	2.0	
No charge	<1	<1		<1	<1	
Other	4.1	3.1		2.8	2.8	
Census division of the hospital			0.065			0.7451
New England	4.0	4.0		5.7	5.1	
Mid-Atlantic	15.2	16.5		16.0	16.2	
East North Central	17.3	16.3		19.2	16.2	
West North Central	7.7	7.3		8.2	7.5	
South Atlantic	21.2	18.5		18.2	18.3	
East South Central	5.8	7.4		6.1	7.1	
West South Central	10.0	11.8		10.0	10.9	
Mountain	7.0	6.0		6.8	8.5	
Pacific	11.7	12.0		9.9	10.3	
Relative bed size category of the hospital			0.0842			0.629
Small	25.6	22.8		22.9	20.9	
Medium	26.3	26.1		27.1	28.0	
Large	48.2	51.1		50.0	51.1	
Location/teaching status of the hospital			0.0981			0.0637
Rural	11.0	9.3		11.5	8.3	
Urban non-teaching	18.0	17.0		16.9	15.6	
Urban teaching	71.0	73.8		71.6	76.1	
Palliative care services	4.3	47.1	<0.0001	4.9	54.2	<0.0001
Charlson comorbidities			<0.0001			<0.0001
0 comorbidities	15.3	4.6		<1	<1	
1 comorbidity	11.6	5.8		<1	<1	
2 comorbidities	23.8	18.8		31.9	20.3	
≥3 comorbidities	49.7	71.0		67.7	79.1	

Comorbidities in leukemic patients with COVID-19

COVID-19 patients with more than three comorbidities had higher mortality rates (p < 0.0001). Several comorbidities were significantly associated with increased mortality. Pulmonary dysfunctions, including acute respiratory distress syndrome (ARDS), acute respiratory failure, acute pulmonary embolism, and ventilator-associated pneumonia, showed higher mortality (p ≤ 0.0001). Renal and metabolic factors, such as acute kidney injury, acidosis, hepatic dysfunction, and acute metabolic encephalopathy, also showed higher mortality (p ≤ 0.0001). Cardiac factors, including arrhythmia, acute heart failure, hypotension, myocardial infarction, cardiogenic shock, and sudden cardiac arrest, were significantly associated with increased mortality (p ≤ 0.0001). Similarly, bone marrow dysfunctions, such as anemia, thrombocytopenia, and pancytopenia, were associated with higher mortality (p ≤ 0.0001). HSCT was not significantly associated with mortality (p > 0.05). Interventions such as hemodialysis, cardiopulmonary resuscitation, invasive ventilation, tracheostomy, and vasopressor usage were common (p < 0.05). Table 2 shows the comorbidities and complications leading to mortality in leukemia patients with COVID-19.

**Table 2:** Comorbidities and complications in leukemia and lymphoma patients with COVID-19. * Arrhythmia included ventricular fibrillation, ventricular tachycardia, atrial fibrillation, and atrial flutter. ARDS: acute respiratory distress syndrome; HSCT: hematopoietic stem cell transplantation; CPR: cardiopulmonary resuscitation.

	Leukemic patients with COVID-19			Lymphoma patients with COVID-19		
Pulmonary Variables	Survival percentage (%) (n = 6991)	Mortality percentage (%) (n = 1200)	p-value	Survival percentage (%) (n = 2071)	Mortality percentage (%) (n = 507)	p-value
Acute respiratory failure	61.5	64.8	0.0296	55.2	66.3	<0.0001
ARDS	4.6	30.2	<0.0001	3.0	30.4	<0.0001
Acute pulmonary embolism	<1	<1	0.1877	<1	<1	0.0613
Renal and metabolic variables						
Acute kidney injury	22.4	57.5	<0.0001	21.3	53.5	<0.0001
Acidosis	8.6	29.1	<0.0001	6.5	26.0	<0.0001
Hemodialysis	3.2	12.1	<0.0001	1.8	9.7	<0.0001
Hepatic dysfunction	0.3	4.6	<0.0001	<1	3.4	<0.0001
Metabolic encephalopathy	4.9	12.8	<0.0001	4.2	11.8	<0.0001
Diabetes	33.0	37.3	0.003	30.0	30.6	0.8129
Cardiovascular variables						
Arrhythmia	9.0	18.2	<0.0001	9.0	18.2	<0.0001
Acute heart failure	8.2	16.0	<0.0001	8.2	16.0	<0.0001
Stroke	0.3	1.4	0.0019	<1	1.4	0.0019
Hypotension	7.3	12.6	0.0001	7.3	12.6	0.0001
Myocardial infarction	4.6	3.6	<0.0001	4.6	3.4	<0.0001
Cardiogenic shock	0.2	1.6	0.0001	<1	1.6	0.0001
Sudden cardiac arrest	0.1	11.8	<0.0001	<1	11.8	<0.0001
Hemato-oncological variables						
Anemia	36.2	45.2	<0.0001	34.7	45.2	<0.0001
Thrombocytopenia	11.5	21.9	<0.0001	13.0	24.7	<0.0001
Neutropenia	3.0	3.0	0.9942	8.2	6.1	0.1227
Pancytopenia	7.5	14.5	<0.0001	11.9	18.2	0.0002
HSCT	4.2	3.7	0.423	4.6	3.4	0.2218
Infections and others						
HIV	0.0	0.0	0.4729	<1	0.0	0.3912
Fungal infections	0.6	1.8	<0.0001	1.3	5.5	<0.0001
Severe sepsis	2.0	25.0	<0.0001	1.6	22.7	<0.0001
ICU-related variables						
Tracheostomy	1.1	2.9	0.0086	0.8	2.2	0.0086
CPR	0.2	11.8	<0.0001	<1	9.5	<0.0001
Invasive ventilation	0.7	9.4	<0.0001	<1	8.9	<0.0001
Ventilator-associated pneumonia	0.2	2.0	<0.0001	<1	2.0	<0.0001
Vasopressor usage	0.5	2.6	<0.0001	<1	15.0	<0.0001

Multivariate regression analysis was performed to determine whether COVID-19 is associated with increased mortality. The results showed that COVID-19 was associated with a two-fold increase in the odds of mortality (p < 0.001). Among leukemia patients with COVID-19, pancytopenia had 80% increased odds (p < 0.001) and thrombocytopenia had 30% increased odds (p < 0.01) of mortality, while neutropenia and anemia (p > 0.05) did not alter mortality. Additionally, there was no difference in mortality in patients who were treated with HSCT (p > 0.05). Table 3 shows multivariate logistic regression for leukemia and lymphoma patients with COVID-19.

**Table 3:** Multivariate logistic regression for leukemia and lymphoma patients with COVID-19. Multivariate analysis was adjusted for age, gender, race, hospital size, hospital teaching status, hypertension, complications (acute respiratory failure, ARDS, severe sepsis, acute kidney injury, myocardial infarction, acute heart failure, arrhythmia, cardiogenic shock, sudden cardiac arrest, and stroke), and interventions (blood transfusion, invasive ventilation, and vasopressor). ARDS: acute respiratory distress syndrome; HSCT: hematopoietic stem cell transplantation.

Leukemia	Adjusted odds ratio (aOR)	95% CI	p-value
COVID-19	2.3	2.0 - 2.7	<0.001
Leukemia patients with COVID-19	Adjusted odds ratio (aOR)	95% CI	p-value
Pancytopenia	1.8	1.4 - 2.3	<0.001
Neutropenia	1.6	0.95 - 2.8	0.08
Thrombocytopenia	1.3	1.1 - 1.6	0.006
Anemia	1.1	0.9 - 1.2	0.59
HSCT	0.75	1.4 - 2.3	0.174
Lymphoma	Adjusted odds ratio (aOR)	95% CI	p-value
COVID-19	2.8	2.2 - 3.6	<0.001
Lymphoma patients with COVID-19	Adjusted odds ratio (aOR)	95% CI	p-value
Pancytopenia	1.6	1.1 - 2.3	0.024
Neutropenia	0.8	0.5 - 1.5	0.529
Thrombocytopenia	1.7	1.2 - 2.4	0.004
Anemia	1.14	0.9 - 1.5	0.331
HSCT	0.43	0.2 - 1.1	0.086

Lymphoma with COVID-19

​​​​Of 2,578 lymphoma patients with COVID-19, 507 had mortality (19.7%). In patients aged 65 years and older, the proportion of non-survivors was significantly higher than that of survivors (p < 0.0001). There was no significant difference in mortality by race and gender (p > 0.05). Table 1 presents the sociodemographic characteristics of lymphoma patients with COVID-19.

Comorbidities in lymphoma patients with COVID-19

COVID-19 patients with more than three comorbidities had higher mortality rates compared to those who survived (p < 0.0001). Several comorbidities were significantly associated with increased mortality. Pulmonary dysfunctions, including ARDS, acute respiratory failure, and ventilator-associated pneumonia, were linked to higher mortality (p ≤ 0.0001). Renal and metabolic factors, such as acute kidney injury, acidosis, hepatic dysfunction, and metabolic encephalopathy, also showed higher mortality (p ≤ 0.0001). Cardiac factors, including arrhythmia, acute heart failure, hypotension, myocardial infarction, and cardiogenic shock, were associated with increased mortality (p ≤ 0.0001). Bone marrow dysfunctions, such as anemia (p ≤ 0.0001), thrombocytopenia (p ≤ 0.0001), and pancytopenia (p < 0.001), were also associated with higher mortality. HSCT was not associated with mortality (p > 0.05). Interventions such as hemodialysis, invasive ventilation, cardiopulmonary resuscitation, tracheostomy, and vasopressor use were required more in those who faced mortality (p < 0.05). Table 2 shows the comorbidities and complications leading to mortality in lymphoma patients with COVID-19.

Multivariate regression analysis was performed to determine whether COVID-19 is associated with increased mortality. The results showed that COVID-19 was associated with a two-fold increase in the odds of mortality (p < 0.001). Of lymphoma patients with COVID-19, pancytopenia had 60% increased odds (p < 0.05) and thrombocytopenia had 70% increased odds (p < 0.01) of mortality, while neutropenia and anemia (p > 0.05) did not alter mortality. Additionally, HSCT did not alter mortality (p > 0.05). Table 3 shows multivariate logistic regression for leukemia and lymphoma patients with COVID-19.

Our study showed that AML showed the highest mortality rate (21.7%), followed by myelodysplastic syndrome (18.6%), chronic myeloid leukemia (CML, 18.5%), multiple myeloma (18.4%), Hodgkin lymphoma (13.5%), leukemia (10.7%), and lymphoma (10.2%). Our analysis of COVID-19 in hematological malignancies from April 2020 to December 2021 revealed significant trends in mortality rates in different quartiles. Leukemia showed more than 15% proportional mortality in the discharge quartile from October to December 2020 (19.1%) and January to March (22.6%), July to September (16.9%), and October to December (16.5%) in 2021 (p < 0.001). Chronic lymphocytic leukemia (CLL) had a significant increase in proportional mortality during October to December (19.9%) in 2020 and January to March (20.1%), July to September (15.6%), and October to December (26.3%) in 2021 (p = 0.001). In AML, CML, multiple myeloma, and myelodysplastic syndrome, proportional mortality did not vary between the different quartiles (p > 0.05).

Lymphoma patients had significant proportional mortality during October to December (16.4%) in 2020 and January to March (24%), July to September (15.5%), and October to December (16.4%) in 2021 (p = 0.01). Hodgkin lymphoma showed no significant proportional mortality increase during these periods (p > 0.05).

**Figure 1:**
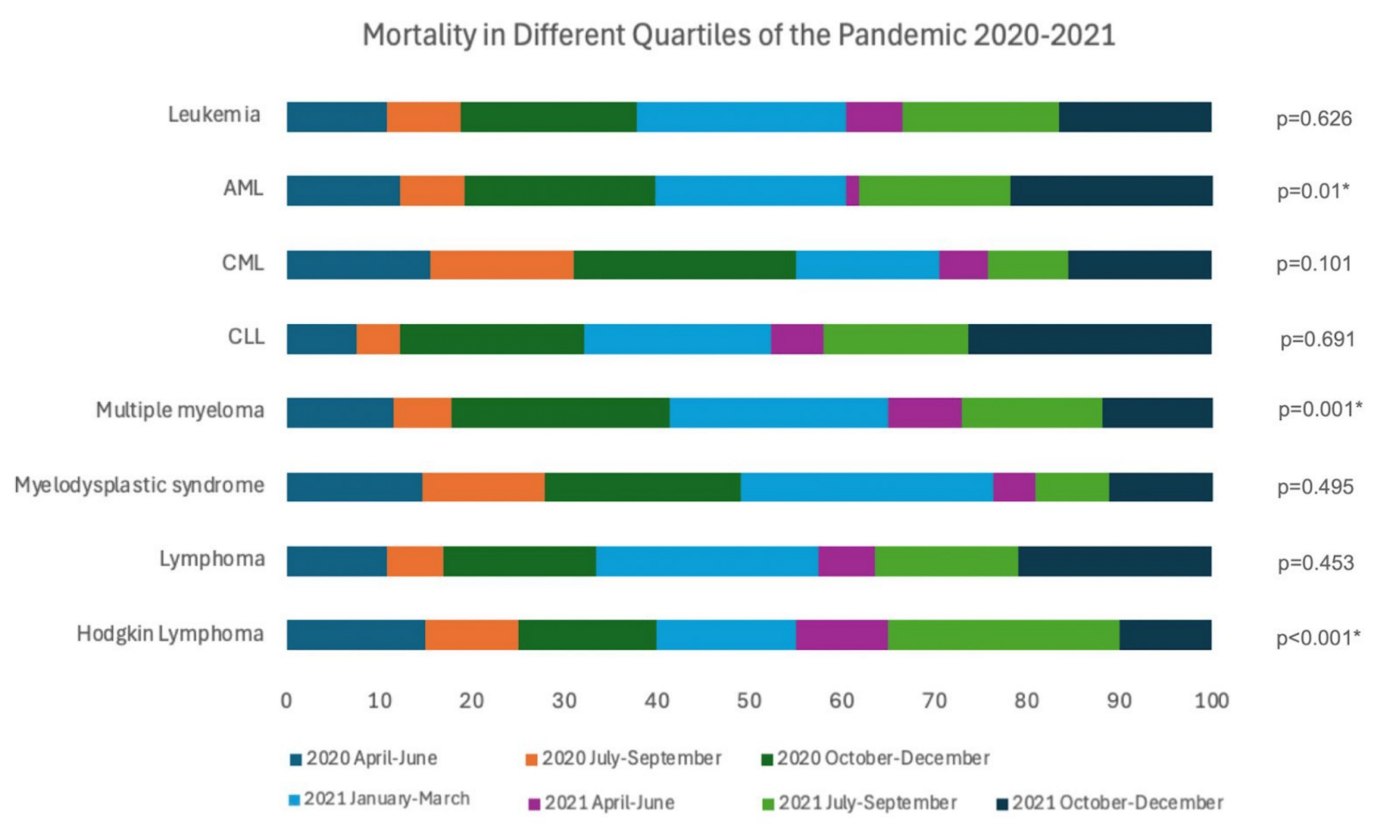
Mortality in different quartiles of the pandemic (2020-2021). * Significant value. AML: acute myeloid leukemia; CML: chronic myeloid leukemia; CLL: chronic lymphocytic leukemia.

These findings emphasize that the October to December 2020 and January to March 2021 quartiles were particularly critical, with all hematological malignancies exhibiting proportional mortality rates exceeding 15%.Supplementary Table A2 shows quarterly trends in COVID-19-related mortality in patients with hematological malignancies (April 2020 - December 2021). Figure 1 shows mortality in different quartiles of the pandemic (2020-2021).

## Discussion

Using a retrospective study design, we identified the mortality risk in hospitalized COVID-19 patients with pre-existing leukemia and lymphoma. In patients with malignancies, COVID-19 has been shown to have a severe presentation due to immune dysfunction leading to low secretion of IFN-1. COVID-19 further compromises immunity by disrupting regulatory pathways such as the adrenergic and immune checkpoint pathways. These mechanisms are also affected in those with malignancies, amplifying immune suppression and increasing the risk of severe outcomes and mortality [24]. Our analysis showed that COVID-19 was associated with a two-fold increase in the odds of mortality in leukemia and lymphoma patients (p < 0.001). In our study, thrombocytopenia and pancytopenia were associated with increased odds of mortality in both cohorts (p < 0.01). Studies indicate that COVID-19 significantly impacts the hematopoietic system, causing cytopenias (lymphopenia, thrombocytopenia, leukopenia) and hypercoagulopathy [25]. Thrombocytopenia may result from decreased platelet production, increased destruction, sequestration, or consumption [25]. Pancytopenia in COVID-19 may result from molecular mimicry, cytokine-mediated suppression of hematopoiesis, and viral bone marrow infiltration [25,26]. HSCT recipients had lower odds of mortality in both cohorts (p > 0.05), although this difference did not reach statistical significance. This may be attributable to the inability to account for the timing of HSCT and the immune status of the patients. This finding is consistent with the study by Karatas et al., which suggested that mortality rates among HSCT recipients are lower in patients whose primary disease is in remission, compared to those whose disease is not in remission [27].

We found that COVID-19 mainly affects the elderly, and Sharafeldin et al. reported age ≥ 65 years (HR: 1.9, 95% CI: 1.3- 3.1) as one of the risk factors for death in cancer patients [19,28,29]. Immunosenescence, deteriorating immunity with progressing age, makes older individuals susceptible to adverse outcomes [30]. Sepsis and fungal infections are significant factors associated with mortality (p < 0.001). Secondary infections and multiorgan failure are known outcomes of COVID-19 [31-33]. COVID-19 is associated with high mortality in patients with hematological malignancies who develop ARDS [13,34]. The pathogenesis is a consequence of increased neutrophilic and eosinophilic activity and high cytokine release (IL-6, TNF-ɑ) [35]. Invasive ventilation is commonly associated with increased mortality (p < 0.001). Studies have shown that mechanical ventilation was associated with a four-fold rise in mortality (p = 0.02) [15,36]. Ventilated patients are also at risk of secondary infections, pneumothorax, and asphyxia due to ventilator-associated complications [37], which may explain the higher mortality associated with ventilator-associated pneumonia observed in our study (p < 0.001). Acute kidney injury from COVID-19 is due to direct invasion of podocytes and proximal tubular cells, leading to acute tubular injury, tubulointerstitial injury, and glomerular injury. Other factors contributing to renal damage include severe volume depletion, cytokine storm, collapsing glomerulopathy, and secondary hypoxia of renal vascular endothelium due to elevated IL-6 [38,39]. COVID-19 has high cardiac complications such as arrhythmia, acute heart failure, myocardial infarction, and cardiogenic shock (p < 0.001), as shown in our study. COVID-19 can increase the risk of thrombosis and directly damage myocardial cells, leading to cardiomyopathy and acute heart failure [40-42].

Our study examined COVID-19 mortality from April 2020 to December 2021 in patients with hematological malignancies and noted trends aligning with shifts in SARS-CoV-2. Mortality rates rose during the April-June 2020 quartile, coinciding with the spread of the wild-type strain [26,43,44]. Before April, COVID-19 testing was not widely used. After June, a slowing of cases was seen in July-September 2020, likely attributable to mask mandates and additional restrictions implemented across various states that helped control transmission. However, as winter approached, the October-December 2020 and January-March 2021 quartiles became particularly critical, with all hematological malignancies exhibiting high mortality rates [26,43-45]. During the April-June 2021 quartile, a drop in mortality rates was seen, likely due to increased vaccination coverage that began in December 2020. However, mortality rates rose starting in July 2021, coinciding with the spread of the more transmissible and severe Delta variant [46]. The emergence of Omicron BA.1 in December 2021 and reduced vaccine effectiveness in cancer patients and their greater risk of severe COVID-19 further contributed to the surge in cases [26,47,48]. Wood et al. reported that the mortality of CLL patients with COVID-19 was 28%, compared to 17.9% in our study [49]. The differences could be due to the different sample sizes in both studies. CLL patients may not consistently generate anti-SARS-CoV-2 antibodies [50], leading to vaccine inefficiency and poorer outcomes. In our study, CLL patients were also noted to have high mortality in the last quartile of 2021, when all other malignancies had lower rates.

This study was limited by the absence of follow-up and information regarding the staging of the malignancies, treatment, treatment responses, and vaccine status. Consequently, we are unable to determine the total effect of vaccines and their side effects. Additionally, as our sample included individuals admitted for COVID-19, we could not estimate the overall impact of COVID-19, at-home mortality rates, or post-discharge outcomes. The underrepresentation of racial minorities leads to inconclusive evidence when it comes to racial disparities. Duplication of patient data and coding errors from the use of ICD-10 were not addressed. The database only includes information from the United States, so its findings may not apply to other countries. The database does not include the exact lab values, which also limits our understanding of the comorbidities in greater detail.

## Conclusions

This study highlights the increased mortality associated with COVID-19 among leukemia and lymphoma patients, with pancytopenia and thrombocytopenia linked to higher mortality in these cohorts. Mortality was highest between October 2020 and March 2021. Understanding these mortality trends from the COVID-19 pandemic is crucial for forecasting future mortality risks in cancer patients. As vaccination and antiviral therapies have evolved, it is important to examine how these interventions have influenced outcomes in patients with hematologic malignancies. Research should also prioritize refining treatment regimens, strengthening preventive strategies, advancing novel therapies, and ensuring long-term follow-up care for this vulnerable population.

## Appendices

Supplementary Table A1

**Table 4:** ICD-10 codes. ICD-10: International Classification of Diseases, Tenth Revision; AML: acute myeloid leukemia; CML: chronic myeloid leukemia; CLL: chronic lymphocytic leukemia; ARDS: acute respiratory distress syndrome; MDS: myelodysplastic syndrome.

Patient characteristics	Codes
Myocardial infarction	I200; I201; I208; I209; I213; I214; I220; I221; I222; I228.x; I240; I241; I248; I249; I2101; I2102; I2109; I2111; I2119; I2121; I2129; Z951; Z9861; Z955; 0270346; 027034Z; 0270446; 027044Z; 0270356; 027035Z; 0270456; 027045Z; 0270366; 027036Z; 0270466; 027046Z; 0270376; 027037Z; 0270476; 027047Z; 0271346; 027134Z; 0271446; 027144Z; 0271356; 027135Z; 0271456; 027145Z; 0271366; 027136Z; 0270376; 0271466; 027146Z; 0271376; 027137Z; 0271476; 027147Z; 0272346; 027234Z; 0272446; 027244Z; 0272356; 027235Z; 0272456; 027245Z; 0272366; 027236Z; 027246Z; 0272376; 027237Z; 0272476; 027035Z; 027247Z; 0273346; 027334Z; 0273446; 027344Z; 0273356; 027335Z; 0273456; 027345Z; 0273366; 027336Z; 0273466; 027346Z; 0273376; 027337Z; 027045Z; 0273476; 027347Z; 02703D6; 02703DZ; 02704D6; 02704DZ; 02703E6; 02703EZ; 02704E6; 02704EZ; 02703F6; 02703FZ; 02704F6; 02704FZ; 02703G6; 02703GZ; 02704G6; 02704GZ; 02713D6; 02713DZ; 02714D6; 02714DZ; 02713E6; 02713EZ; 02714E6; 02714EZ; 02713F6; 02713FZ; 02714F6; 02714FZ; 02713G6; 02713GZ; 02714G6; 02714GZ; 02723D6; 02723DZ; 02724D6; 02724DZ; 02723E6; 02723EZ; 02724E6; 02724E6; 02724EZ; 02723F6; 02723FZ; 02724F6; 02724FZ; 02723G6; 02723GZ; 02724G6; 02724GZ; 02733D6; 02733DZ; 02734D6; 02734DZ; 02733E6; 02733EZ; 02734E6; 02734EZ; 02733F6; 02733FZ; 02733FZ; 02734F6; 02733G6; 02733GZ; 02734G6; 02734GZ; 02703ZZ; 02704ZZ; 02713ZZ; 02714ZZ; 02723ZZ; 02724ZZ; 02733ZZ; 02734ZZ; 0210093; 0210098; 0210099; 021009C; 021009F; 021009W; 02100A3; 02100A8; 02100A9; 02100AC; 02100AF; 02100AW; 0211093; 0211098; 0211099; 021109C; 021109F; 021109W; 02110A3; 02110A8; 02110A9; 02110AC; 02110AF; 02110A; 0212093; 0212098; 0212099; 021209C; 021209F; 021209W; 02120A3; 02120A8; 02120A9; 02120AC; 02120AF; 02120AW; 0213093; 0213098; 0213099; 021309C; 021309F; 021309W; 02130A3; 02130A8; 02130A9; 02130AC; 02130AF; 02130AW
HIV	C220; C227; C228; C229
Opportunistic infections	B371; B3781; B380; B381; B382; B383; B384; B387; B388; B3881; B3889; B389; B389; B451; B452; B453; B457; B458; B459; A072; B250; B251; B252; B258; B259; B393; B394; B399; A073; A311; A312; A318; A319; A15; A150; A154.x; A17; A170; A171; A178; A179; A18; A180.x; A19; A190; A191; A192; A198; A199; B59; B582; B390.x; B39; B59; B440; B441; B442; B447; B448; B4481; B4489; B449; B4081; B4089; B409; B400; B401; B402; B403; B407; B450; B451; B452; B453; B457; B458; B459
Blood transfusion	30233N1; 30243N; 30243P0; 30233P0
Vasopressor	3E050XZ; 3E063XZ; 3E060XZ; 3E053XZ; 3E043XZ; 3E040XZ; 3E033XZ; 3E030XZ
Respiratory failure	J9620; J9621; J9622; J9600; J9601; J9602
ARDS	J80
Sepsis	R6520; R6521
Acute kidney injury	N170; N171; N172; N178; N179
Arrhythmias	I4901; I472; I480; I481; I482; I483; I484; I491; I492
Cardiogenic shock	R570
Sudden cardiac arrest	I462; I468; I469
Acute heart failure	I5040; I5041; I5043; I509; I5030; I5031; I5033; I5023; I5020; I5021; I501; J810
AML	C9200; C9201; C9202; C9252; C9240; C9260; C9261; C9262; C92A0; C92A1; C92A2; C9250; C9251; C9252; C9240; C9241; C9242; C9300; C9301; C9302; С9330; C9331; C9332
MDS	D464; D469; D46A; D46B; D46C; D46Z; D4620; D4621; D4622; D460; D461
Stroke	I63; I6050; I6051; I6052; I606; I607; I608; I609; I6030; I6031; I6032; I604; I6010; I6011; I6012; I602; I600; I601; I602; I610.x; I618; I619; I6200; I6201; I6202; I6203; I621; I629; I6300; I6301; I6302; I6303; I631; I629; I6300; I6301; I6302; I6303; I6309; I6310; I6311; I6312; I6313; I6319; I6320; I6321; I6322; I6323; I6329; I6330; I6331; I6332; I6333; I6339; I6340; I6341; I6342; I6343; I6349; I6350; I6351; I6352; I6353; I6359; I6360; I6361; I6362; I6363; I6369; I6370; I6371; I6372; I6373; I6379; I6380; I6381; I6382; I6383; I6389; I6390; I6391; I6392; I6393; I6399; I640; I641; I6411; I6412; I6413; I6419; I642; I6421; I6422; I6423; I6429; I643; I6431; I6432; I6433; I6439; I644; I6441; I6442; I6443; I6449; I645; I6451; I6452; I6453; I6459; I646; I6461; I6462; I6463; I6469; I647; I648; I6481; I6482; I6483; I6489; I649; I6491; I6492; I6493; I6499; I650; I651; I652; I660; I670; I680; I690
Diabetes	E0800; E0801; E0810; E0811; E0821; E0822; E0829; E08311; E08319; E08321; E083211; E083212; E083213; E083219; E08329; E083291; E083292; E083293; E083299; E08331; E083311; E083312; E083313; E083319; E08339; E083391; E083392; E083393; E083399; E08341; E083411; E083412; E083413; E083419; E08349; E083491; E083492; E083493; E083499; E08351; E083511; E083512; E083513; E083519; E083521; E083522; E083522; E083529; E083531; E083532; E083533; E083539; E083541; E083542; E083543; E083549; E083551; E083552; E083553; E083559; E08359; E083591; E083592; E083593; E083599; E0836; E0837X1; E0837X2; E0837X3; E0837X9; E0839.x; E0849; E0851; E0852; E0859; E08610; E08618; E08620; E08621; E08622; E08628; E08630; E08638; E08641; E08649; E0865; E0869; E088; E089; E0900; E0901; E0910; E0911; E0921; E0922; E0929; E09311; E09319; E09321; E093211; E093212; E093213; E093219; E09329; E093291; E093292; E093293; E093299; E09331; E093311; E093312; E093313; E093319; E09339; E093391; E093392; E093393; E093399; E09341; E093411; E093412; E093413; E093419; E09349; E093491; E093492; E093493; E093499; E09351; E093511; E093512; E093513; E093519; E093521; E093522; E093523; E093529; E093531; E093532; E093533; E093539; E093541; E093542; E093543; E093549; E093551; E093552; E093553; E093559; E09359; E093591; E093592; E093593; E093599; E0936; E0937X1; E0937X2; E0937X3; E0937X9; E0939.x; E0949; E0951; E0952; E0959; E09610; E09618; E09620; E09621; E09622; E09628; E09630; E09638; E09641; E09649; E0965; E0969; E098; E099; E1010; E1011; E1021; E1022; E1029; E10311; E10319; E10321; E103211; E103212; E103213; E103219; E10329; E103291; E103292; E103293; E103299; E10331; E103311; E103312; E103313; E103319; E10339; E103391; E103392; E103393; E103399; E10341; E103411; E103412; E103413; E103419; E10349; E103491; E103492; E103493; E103499; E10351; E103511; E103512; E103513; E103519; E103521; E103522; E103523; E103529; E103531; E103532; E103533; E103539; E103541; E103542; E103543; E103549; E103551; E103552; E103553; E103559; E10359; E103591; E103592; E103593; E103599; E1036; E1037X1; E1037X2; E1037X3; E1037X9; E1039; E1040; E1041; E1042; E1043; E1044; E1049; E1051; E1052; E1059; E10610; E10618; E10620; E10621; E10622; E10628; E10630; E10638; E10641; E10649; E1065; E1069; E108; E109; E1100; E1101; E1110; E1111; E1121; E1122; E1129; E11311; E11319; E11321; E113211; E113212; E113213; E113219; E11329; E113291; E113292; E113293; E113299; E11331; E113311; E113312; E113313; E113319; E11339; E113391; E113392; E113393; E113399; E11341; E113411; E113412; E113413; E113419; E11349E; 113491; E113492; E113493; E113499; E11351; E113511; E113512; E113513; E113519; E113521; E113522; E113523; E113529; E113531; E113532; E113533; E113539; E113541; E113542; E113543; E113549; E113551; E113552; E113553; E113559; E11359; E113591; E113592; E113593; E113599; E1136; E1137X1; E1137X2; E1137X3; E1137X9; E1139; E1140; E1141; E1142; E1143; E1144; E1149; E1151; E1152; E1159; E11610; E11618; E11620; E11621; E11622; E11628; E11630; E11638; E11641; E11649; E1165; E1169; E118; E119; E1300; E1301; E1310; E1311; E1321; E1322; E1329; E13311; E13319; E13321; E133211; E133212; E133213; E133219; E13329; E133291; E133292; E133293; E133299; E13331; E133311; E133312; E133313; E133319; E13339; E133391; E133392; E133393; E133399; E13341; E133411; E133412; E133413; E133419; E13349; E133491; E133492; E133493; E133499; E13351; E133511; E133512; E133513; E133519; E133521; E133522; E133523; E133529; E133531; E133532; E133533; E133539; E133541; E133542; E133543; E133549; E133551; E133552; E133553; E133559; E13359; E133591; E133592; E133593; E133599; E1336; E1337X1; E1337X2; E1337X3; E1337X9; E1339; E1340; E1341; E1342; E1343; E1344; E1349; E1351; E1352; E1359; E13610; E13618; E13620; E13621; E13622; E13628; E13630; E13638; E13641; E13649; E1365; E1369; E138; E139
COVID-19	U071
Hodgkin	C8194; C8195; C8196; C8197; C8198; C8199; C8190; C8191; C8192; C8193; C8170; C8171; C8172; C8173; C8174; C8175; C8176; C8177; C8178; C8179; C8140; C8141; C8142; C8143; C8144; C8145; C8146; C8147; C8148; C8149; C8130; C8131; C8132; C8133; C8134; C8135; C8136; C8137; C8138; C8139; C8120; C8121; C8122; C8123; C8110; C8111; C8112; C8113; C8114; C8115; C8116; C8117; C8118; C8119; C8100; C8101; C8102; C8103; C8104; C8105; C8106; C8107; C8108; C8109
Lymphoma	C8194; C8195; C8196; C8197; C8198; C8199; C8190; C8191; C8192; C8193; C8170; C8171; C8172; C8173; C8174; C8175; C8176; C8177; C8178; C8179; C8140; C8141; C8142; C8143; C8144; C8145; C8146; C8147; C8148; C8149; C8130; C8131; C8132; C8133; C8134; C8135; C8136; C8137; C8138; C8139; C8120; C8121; C8122; C8123; C8110; C8111; C8112; C8113; C8114; C8115; C8116; C8117; C8118; C8119; C8100; C8101; C8102; C8103; C8104; C8105; C8106; C8107; C8108; C8109; C8293; C8294; C8295; C8296; C8297; C8298; C8299; C8290; C8291; C8292; C8284; C8285; C8286; C8287; C8288; C8289; C8280; C8281; C8282; C8283; C8260; C8261; C8262; C8263; C8264; C8265; C8266; C8267; C8268; C8269; C8254; C8255; C8256; C8257; C8258; C8259; C8250; C8251; C8252; C8253; C8243; C8244; C8245; C8246; C8247; C8248; C8249; C8240; C8241; C8242; C8233; C8234; C8235; C8236; C8237; C8238; C8239; C8230; C8231; C8232; C8224; C8225; C8226; C8227; C8228; C8229; C8220; C8221; C8222; C8223; C8210; C8211; C8212; C8213; C8214; C8215; C8216; C8217; C8218; C8219; C8200; C8201; C8202; C8203; C8204; C8205; C8206; C8207; C8208; C8209; C8390; C8391; C8392; C8393; C8394; C8395; C8396; C8397; C8398; C8399; C8380; C8381; C8382; C8383; C8384; C8385; C8386; C8386; C8387; C8388; C8389; C8372; C8373; C8374; C8375; C8376; C8377; C8378; C8379; C8370; C8371; C8372; C8354; C8355; C8356; C8357; C8358; C8359; C8350; C8351; C8352; C8353; C8333; C8334; C8335; C8336; C8337; C8338; C8339; C8330; C8331; C8332; C8333; C8310; C8311; C8312; C8313; C8314; C8315; C8316; C8317; C8318; C8319; C8303; C8304; C8305; C8306; C8307; C8308; C8309; C8300; C8301; C8302; C84Z4; C84Z5; C84Z6; C8427; C8428; C8429; C84Z0; C84Z1; C8422; C84Z3; C84Z4; C84A0; C84A1; C84A2; C84A3; C84A4; C84A5; C84A6; C84A7; C84A8; C84A9; C8490; C8491; C8492; C8493; C8494; C8495; C8496; C8497; C8498; C8499; C8474; C8475; C8476; C8477; C8478; C8479; C8470; C8471; C8472; C8473; C8474; C8460; C8461; C8462; C8463; C8464; C8465; C8466; C8467; C8468; C8469; C8440; C8441; C8442; C8443; C8444; C8445; C8446; C8447; C8448; C8449; C8410; C8411; C8412; C8413; C8414; C8415; C8416; C8417; C8418; C8419; C8400; C8401; C8402; C8403; C8404; C8405; C8406; C8407; C8408; C8409; C8594; C8595; C8596; C8597; C8598; C8599; C8590; C8591; C8592; C8593; C8594; C8585; C8586; C8587; C8588; C8589; C8580; C8581; C8582; C8583; C8584; C8524; C8525; C8526; C8527; C8528; C8529; C8520; C8521; C8522; C8523; C8513; C8514; C8515; C8516; C8517; C8518; C8519; C8510; C8511; C8512; C8513; C860; C861; C862; C863; C864; C865; C866; C880; C882; C883; C884; C888; C889
CLL	C9110; C9111; C9112
CML	C9210; C9211; C9212; C9220; C9221; C9222; C9310; C9311; C9312
AML	C9200; C9201; C9202; C9252; C9240; C9260; C9261; C9262; C92A0; C92A1; C92A2; C9250; C9251; C9252; C9240; C9241; C9242; C9300; C9301; C9302; С9330; C9331; C9332
Leukemia	C9100; C9101; C9102; C9110; C9111; C9112; C9130; C9131; C9132; C9140; C9141; C9142; C9150; C9151; C9152; C9160; C9161; C9162; C9190; C9191; C9192; C91A0; C91A1; C91A2; C91Z0; C91Z1; C91Z2; C9200; C9201; C9202; C9210; C9211; C9212; C9220; C9221; C9222; C9230; C9231; C9232; C9240; C9241; C9242; C9250; C9251; C9252; C9260; C9261; C9262; C9290; C9291; C9292; C92A0; C92A1; C92A2; C92Z0; C92Z1; C92Z2; C9300; C9301; C9302; C9310; C9311; C9312; C9330; C9331; C9332; C9390; C9391; C9392; C93Z0; C93Z1; C93Z2; C9400; C9401; C9402; C9420; C9421; C9422; C9430; C9431; C9432; C9440; C9441; C9442; C946; C9480; C9481; C9482; C9500; C9501; C9502; C9510; C9511; C9512; C9590; C9591; C9592; D7581; 2386; 2030; 20300; 20301; 20302; 2038; 20380; 20381; 20382; 2731; 2733; E8581; D473; C9000; C9001; C9002; C9010; C9011; C9012; C9020; C9021; C9022; C9030; C9031; C9032; C880
Stroke	I63; I6050; I6051; I6052; I606; I607; I608; I609; I6030; I6031; I6032; I604; I6010; I6011; I6012; I602; I600; I601; I602; I610; I611; I612; I613; I614; I615; I616; I618; I619; I6200; I6201; I6202; I6203; I621; I629; I6300; I6301; I6302; I6303; I631; I629; I6300; I6301; I6302; I6303; I6309; I6310; I6311; I6312; I6313; I6319; I6320; I6321; I6322; I6323; I6329; I6330; I6331; I6332; I6333; I6339; I6340; I6341; I6342; I6343; I6349; I6350; I6351; I6352; I6353; I6359; I6360; I6361; I6362; I6363; I6369; I6370; I6371; I6372; I6373; I6379; I6380; I6381; I6382; I6383; I6389; I6390; I6391; I6392; I6393; I6399; I640; I641; I6411; I6412; I6413; I6419; I642; I6421; I6422; I6423; I6429; I643; I6431; I6432; I6433; I6439; I644; I6441; I6442; I6443; I6449; I645; I6451; I6452; I6453; I6459; I646; I6461; I6462; I6463; I6469; I647; I648; I6481; I6482; I6483; I6489; I649; I6491; I6492; I6493; I6499; I650; I651; I652; I660; I670; I680; I690

Supplementary Table A2

**Table 5:** Quarterly trends in COVID-19-related mortality among patients with hematological malignancies (April 2020 - December 2021). AML: acute myeloid leukemia; CML: chronic myeloid leukemia; CLL: chronic lymphocytic leukemia

	Leukemia	AML	CML	CLL	Multiple myeloma	Myelodysplastic syndrome	Lymphoma	Hodgkin lymphoma
Total mortality % (2020-2021)	10.7	21.7	18.5	17.9	18.4	18.6	10.2	13.5
p-value	<0.001	<0.001	<0.001	<0.001	<0.001	<0.001	<0.001	<0.001
Mortality % in 2020								
April-June	10.8	12.3	15.5	7.6	11.6	14.6	10.9	15
July-September	8.0	6.9	15.5	4.7	6.2	13.3	6.1	10
October-December	19.1	20.6	24.1	19.9	23.6	21.2	16.4	15
Mortality % in 2021								
January-March	22.6	20.6	15.5	20.1	23.6	27.2	24	15
April-June	6.1	1.4	5.2	5.7	8	4.6	6.1	10
July-September	16.9	16.4	8.6	15.6	15.2	8	15.5	25
October-December	16.5	21.9	15.5	26.3	12	11.3	21	10
p-value	<0.001	0.453	0.495	0.001	0.691	0.101	0.01	0.626
